# High-resolution three-dimensional ANGIE T1 mapping of the whole heart

**DOI:** 10.1186/1532-429X-17-S1-W4

**Published:** 2015-02-03

**Authors:** Bhairav B Mehta, Michael Salerno, Frederick H Epstein

**Affiliations:** 1 Department of Biomedical Engineering, University of Virginia, Charlottesville, VA, USA; 2 Medicine (Cardiology), University of Virginia, Charlottesville, VA, USA; 3 Department of Radiology and Medical Imaging, University of Virginia, Charlottesville, VA, USA

## Background

Assessment of fibrosis in thinner myocardial structures such as the right ventricular (RV) and left atrial walls would be valuable in disorders such as pulmonary hypertension, congenital heart disease, and atrial fibrillation. We recently developed a novel technique, termed ANGIE, which provides high-resolution 2D T1 mapping for assessment of thin structures such as the wall of the RV. However, high resolution 2D imaging is limited by lower SNR and time efficiency. A 3D image acquisition provides higher SNR and complete coverage of the heart. However standard 3D navigated techniques have prohibitively long scan times. Thus, the aim of the present study was to extend ANGIE to perform high-resolution three-dimensional (3D) T1 mapping of the whole heart within a clinically acceptable scan time.

## Methods

A novel image reconstruction algorithm, which uses compressed sensing (CS) with local low-rank sparsity in conjunction with partial Fourier and parallel imaging, was developed to achieve high acceleration (R=10) for 3D ANGIE. High-resolution (1.4x1.4x4mm^3^) T1 mapping of the whole heart was performed using 3D ANGIE in three healthy volunteers on a 1.5T system (Avanto Siemens). Imaging parameters included: matrix size=224x224x26, number of inversion times=12, and partial Fourier reduction factor of 3/4 along phase and partition encode. Additionally, 2D MOLLI (2.5x2x4mm^3^) and 2D ANGIE (1.3x1.3x4mm^3^) scans were performed at three different short-axis slice positions.

## Results

**Figure 1:**
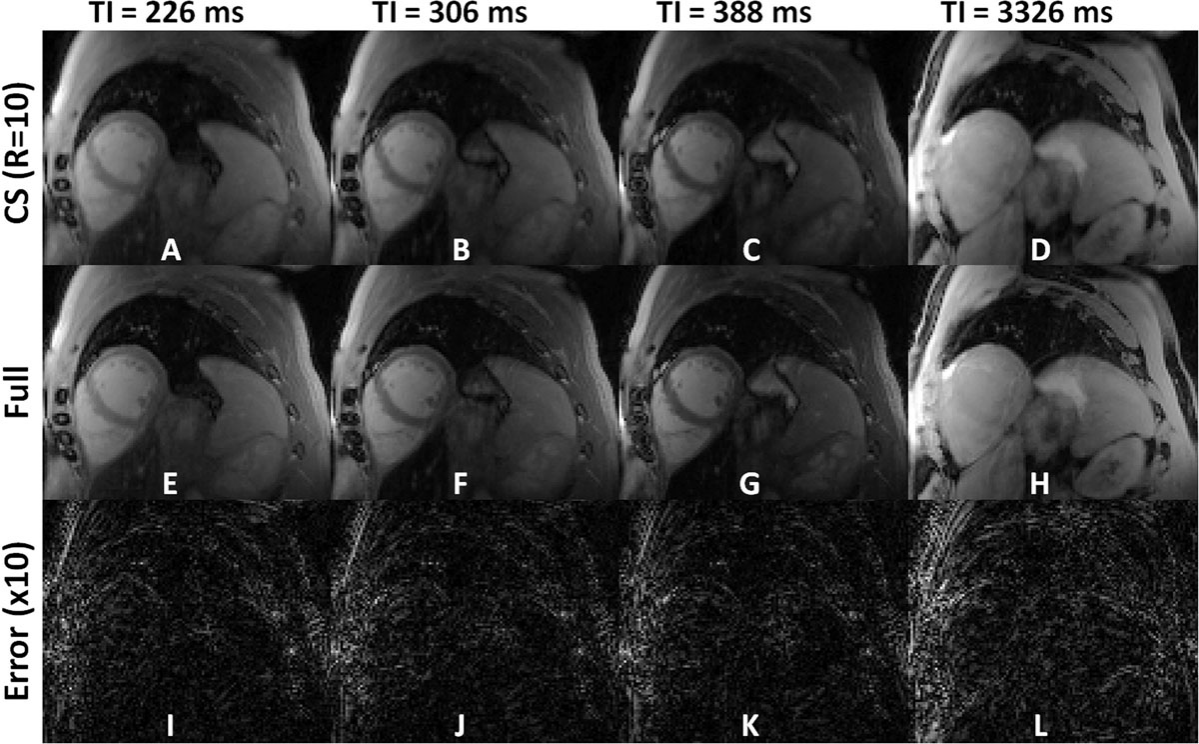
Example reconstruction results from a retrospectively undersampled (rate 10) fully sampled 3D ANGIE dataset. Reconstructed (A-D), fully sampled (E-H) and corresponding reconstruction error (I-L) images from a single partition at four inversion time points of a 3D ANGIE dataset acquired from a healthy volunteer. The image reconstruction algorithm effectively suppresses aliasing artifacts and retains image spatial resolution. These results illustrate the capability of achieving high acceleration rate (R=10) using the proposed image reconstruction algorithm.

**Figure 2:**
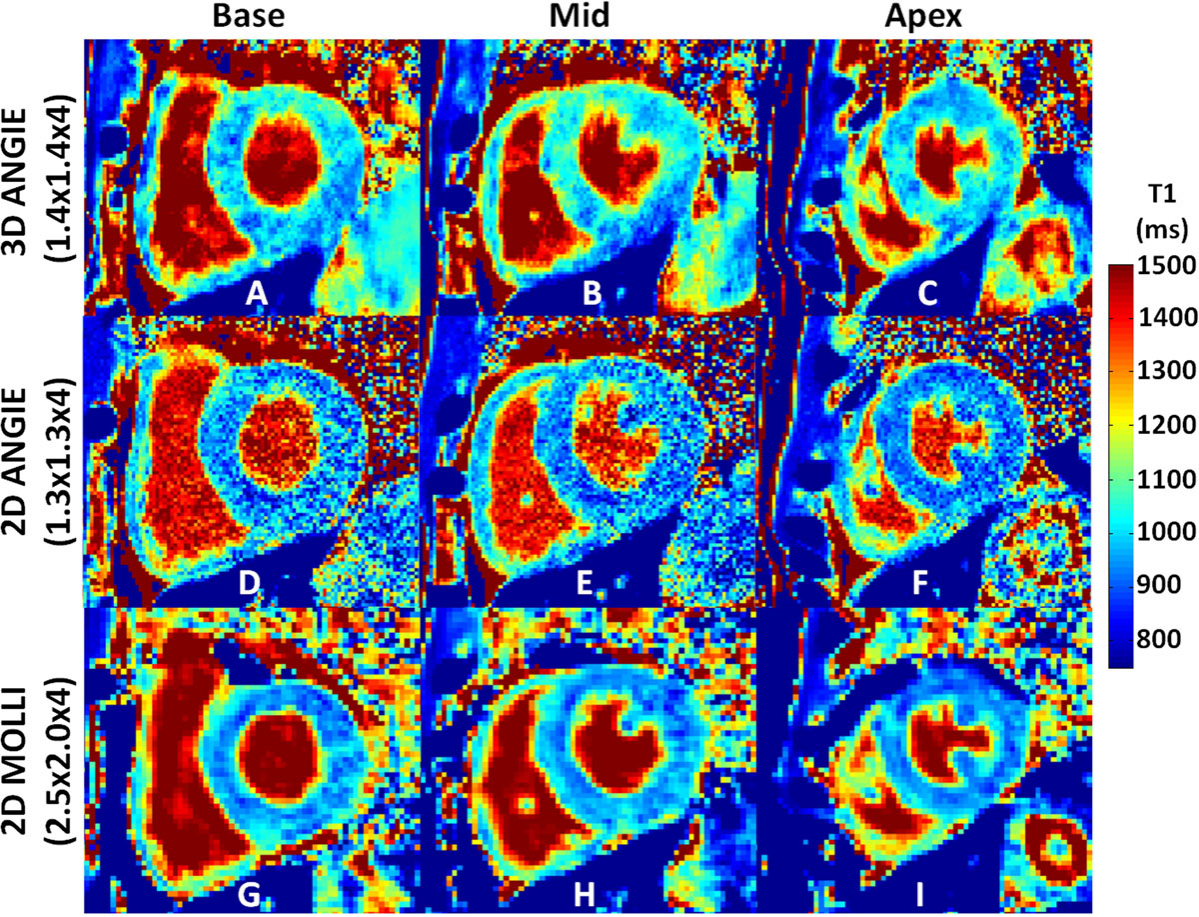
Comparison of T1 mapping techniques in healthy volunteer. Example native T1 maps from a healthy volunteer at three different slice positions acquired using 3D ANGIE (A-C), 2D ANGIE (D-F) and 2D MOLLI (G-I). 2D MOLLI provides high SNR but incomplete definition of the RV wall, whereas 2D ANGIE provides good definition of the RV wall but lower SNR. However, 3D ANGIE provides both good definition of the RV wall as well as high SNR.

Figure 1 illustrates example reconstructed (A-D), fully sampled (E-H) and reconstruction error (I-L) images from a retrospectively accelerated (R=10) 3D ANGIE dataset. The image reconstruction algorithm effectively suppresses aliasing artifacts and retains spatial resolution, illustrating the capability of achieving a high acceleration rate (R=10) using the proposed reconstruction algorithm. Figure 2 illustrates example T1 maps from a healthy volunteer acquired using all three techniques with prospective acceleration at three different slice positions across the ventricle. 2D MOLLI (Fig. 2G-I) provides high SNR but incomplete definition of the RV wall, whereas 2D ANGIE (Fig. 2D-F) provides good definition of the RV wall but lower SNR. However, 3D ANGIE (Fig. 2A-C) provides both good definition of the RV wall as well as high SNR. The left ventricular myocardium and blood T1 estimates of 3D ANGIE (LV: 1013±67ms, blood: 1501±44ms) were in close agreement with 2D MOLLI (LV: 994±81ms, blood: 1507±68ms). The RV T1 estimates of 3D ANGIE (1022±85ms) were in close agreement with 2D ANGIE (1028±89ms). The acquisition time and navigator acceptance for 3D ANGIE was 6.6±2.7mins and 67±19%, while for 2D ANGIE was 2.2± 1.2mins (per slice) and 60±16%.

## Conclusions

3D ANGIE provides accurate high-resolution (1.4x1.4x4mm^3^) native T1 maps of the whole heart within a clinically acceptable scan time.

## Funding

NIH R01 EB 001763, AHA Grant-in-Aid 12GRNT12050301, AHA 14PRE20210008, and Siemens Medical Solutions.

